# Adiposity Is Associated with Blunted Cardiovascular, Neuroendocrine and Cognitive Responses to Acute Mental Stress

**DOI:** 10.1371/journal.pone.0039143

**Published:** 2012-06-20

**Authors:** Alexander Jones, Merlin R. McMillan, Russell W. Jones, Grzegorz T. Kowalik, Jennifer A. Steeden, John E. Deanfield, Jens C. Pruessner, Andrew M. Taylor, Vivek Muthurangu

**Affiliations:** 1 Centre for Cardiovascular Imaging, UCL Institute of Cardiovascular Science, London, United Kingdom; 2 Chorleywood Health Centre, Chorleywood, United Kingdom; 3 Department of Information Systems and Computing, Brunel University, Uxbridge, United Kingdom; 4 Centre for Cardiovascular Prevention & Outcomes, UCL Institute of Cardiovascular Science, London, United Kingdom; 5 Douglas Institute, Department of Psychiatry, McGill University, Montreal, Canada; University of Virginia Health System, United States of America

## Abstract

Obesity and mental stress are potent risk factors for cardiovascular disease but their relationship with each other is unclear. Resilience to stress may differ according to adiposity. Early studies that addressed this are difficult to interpret due to conflicting findings and limited methods. Recent advances in assessment of cardiovascular stress responses and of fat distribution allow accurate assessment of associations between adiposity and stress responsiveness. We measured responses to the Montreal Imaging Stress Task in healthy men (N = 43) and women (N = 45) with a wide range of BMIs. Heart rate (HR) and blood pressure (BP) measures were used with novel magnetic resonance measures of stroke volume (SV), cardiac output (CO), total peripheral resistance (TPR) and arterial compliance to assess cardiovascular responses. Salivary cortisol and the number and speed of answers to mathematics problems in the task were used to assess neuroendocrine and cognitive responses, respectively. Visceral and subcutaneous fat was measured using T_2_^*^-IDEAL. Greater BMI was associated with generalised blunting of cardiovascular (HR:*β* = −0.50 bpm.unit^−1^, *P* = 0.009; SV:*β* = −0.33 mL.unit^−1^, *P* = 0.01; CO:*β* = −61 mL.min^−1^.unit^−1^, *P* = 0.002; systolic BP:*β* = −0.41 mmHg.unit^−1^, *P* = 0.01; TPR:*β* = 0.11 WU.unit^−1^, *P* = 0.02), cognitive (correct answers: r = −0.28, *P* = 0.01; time to answer: r = 0.26, *P* = 0.02) and endocrine responses (cortisol: r = −0.25, *P* = 0.04) to stress. These associations were largely determined by visceral adiposity except for those related to cognitive performance, which were determined by both visceral and subcutaneous adiposity. Our findings suggest that adiposity is associated with centrally reduced stress responsiveness. Although this may mitigate some long-term health risks of stress responsiveness, reduced performance under stress may be a more immediate negative consequence.

## Introduction

Obesity is a major global public health problem. It contributes to risk of cardiovascular mortality by increasing dyslipidaemia, type-2 diabetes and hypertension, but also confers risk independently of these factors [1]. This additional risk is unexplained, but could be due to associations between obesity and stress. Stress exposure has been identified as a cause of both obesity [2] and cardiovascular disease [3]. Therefore, vulnerability to stress may be a link between these conditions and can be tested by measuring responses to acute mental stress (AMS). Although there is evidence that exaggerated cardiovascular responses to AMS predict the development of cardiovascular disorders [4], the link between obesity and stress responsiveness remains unclear.

Few studies have addressed this link and their findings are controversial. Most studies have found that obesity is associated with greater cardiovascular responses to AMS [5], [6], [7]. However, recent work showed that surrogate measures of adiposity such as body mass index (BMI) were associated with lower heart rate (HR) responses to AMS [8]. This is surprising, given the positive associations of cardiovascular disease with obesity and stress exposure. Therefore, these findings need confirmation and some important concerns need to be addressed. To date, studies have used limited measures of cardiovascular function (HR & blood pressure; BP), leading to uncertain interpretation of their results. They have also generally used surrogate measures of adiposity that do not accurately reflect fat distribution. This may be significant because visceral fat appears to be responsible for more of the adverse effects of obesity than subcutaneous fat is.

Resolution of the association between adiposity and responsiveness to AMS requires more complete assessment of both cardiovascular stress responses and body fat distribution. It was recently demonstrated that cardiovascular magnetic resonance (MR) could measure the cardiovascular response to AMS reliably and comprehensively [9]. Furthermore, novel and rapid MR fat-water separation methods have been developed that provide accurate measurement of body fat distribution [10]. Using these advanced techniques, this study investigates the associations of adiposity with cardiovascular and neuroendocrine responses to AMS.

## Methods

We studied healthy men (N = 43) and women (N = 45) with a median age of 38 years, recruited from a single primary care research practice (Chorleywood Health Centre, Hertfordshire, UK). Electronic patient records and disease registers were used to select healthy men and women aged between 18 and 45 years, who were free of chronic disease and did not use regular medication. Participants with contraindications for MR imaging were excluded. The Great Ormond Street Hospital/Institute of Child Health Research Ethics Committee approved the study and participants gave written informed consent.

All participants completed questionnaires on their social class, ethnicity, education, and tobacco and alcohol consumption [11]. Habitual physical activity was estimated using the *Baecke short questionnaire for the measurement of habitual physical activity in epidemiological studies* [12]. This validated, repeatable measure has been shown to correspond well with direct measures of physical activity and cardiorespiratory fitness [13]. Stress exposure in the preceding month was assessed with the Perceived Stress Scale [14].

### Montreal Imaging Stress Task (MIST)

Participants underwent the MIST; a mental stressor designed for use during MR imaging. This has been described in detail previously and shown to reliably stimulate cardiovascular and endocrine responses [9]. Briefly, participants used button presses to answer a series of mental arithmetic problems presented on a computer screen. Their performance was shown continuously on the screen by a moving indicator. They were asked to compare this with another indicator that they were told represented the average performance of previous participants. In fact, it showed a random computer-generated performance that was biased to always finish with a high percentage of correct answers. Participants were told that a strong performance was required for useful results. They were unaware that question difficulty and time allowed for answers was varied by the computer to ensure that all participants achieved a similarly poor percentage of correct answers, regardless of competence. The aim was for all participants to believe that they could be judged negatively in comparison to their peers. This was important because reliable stress provocation needs elements of motivated performance, uncontrollability and social evaluation [15].

MR data was acquired on six occasions: once at rest, then four times during stress (at one and three minutes into two consecutive five-minute stress periods) and finally once during a recovery period. The two stress periods were separated by a short (two minute) interval. Median times for these acquisitions relative to the start of the first stress period were -4 (rest), 1, 3, 8, 10 (stress) and 23 (recovery) minutes. Each acquisition took five seconds. After the rest period and before stress began, there was a one-minute practice session, with no scoring, time limits or evaluation. In the interval between stress periods, the investigator told the participants that they were not performing well and that they would need to improve or their data might not be useful. Participants were told to rest after the last stress period. Importantly, upon completion of the protocol, all participants were informed that their poor performance was artificial and that their data was useful.

### Cardiovascular Measures

Oscillometric BP was measured in the non-dominant arm at one-minute intervals. All imaging was performed on a 1.5T MR scanner (Avanto, Siemens Medical Solutions, Erlangen, Germany).

Flow quantification was performed through-plane in a cross-section of the ascending aorta as it passes the bifurcation of the pulmonary arteries using an ECG-gated spiral phase-contrast MR sequence, as described previously [16]. This technique allows images to be acquired within a short breath-hold (∼5 seconds) with a spatial resolution of 1.6×1.6 mm and a temporal resolution of 30 milliseconds.

All images were processed using in-house plug-ins for the open-source software OsiriX (OsiriX Foundation, Geneva, Switzerland). Flow images were manually segmented (using the modulus images) and stroke volume (SV) and CO were measured. Total peripheral resistance (TPR; measured in mmHg.L^−1^.min^−1^, also known as WU) was calculated by dividing the mean BP (MBP) by CO. Compliance was calculated by optimization of a two-element windkessel model, as previously described [17]. Briefly, CO, MBP and TPR were used as inputs to the model. Pulse pressure (PP) was calculated for a series of modelled pressure curves generated using a range of compliance values from 0.1 to 5.0 mL.mmHg^−1^ in increments of 0.01. The compliance value that gave the smallest error between the modelled PP and the true PP was taken to be the true compliance.

### Fat Quantification

**Figure 1:**
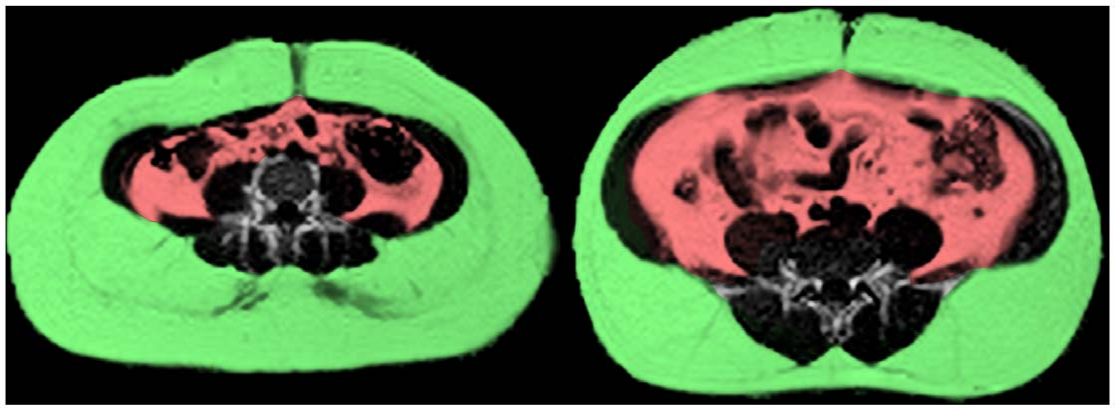
Fat/water ratio images for two participants with the same BMI of 33. Subcutaneous fat is highlighted in green and visceral fat is highlighted in red. Visceral adiposity differs substantially between the two participants (4% on left, 11% on right). This illustrates the advantage of direct fat quantification over surrogate measures of adiposity such as BMI.

We used a graphics processing unit (GPU) implementation of the T_2_^*^-IDEAL algorithm [10], [18] to measure body fat content. This iteratively separates MR images into fat and water components, which can then be used to measure the proportion of fat in each 3×3×10 mm voxel. Data was acquired in a continuous stack of 10 mm thick slices from the neck to the knees. To prevent motion artefact, we used breath holding for the thorax and abdomen and cardiac gating for slices containing the heart. Fat quantification in the head, arms and below the knees was impractical due to the need for participant re-positioning or specialized coils. Due to their low fat content, we excluded these body parts using anatomical landmarks to ensure consistency between participants. The visceral compartment was manually separated from the fat image (Figure 1) and the liver was excluded due to frequent artefacts at diaphragm level.

### Salivary Cortisol

Using established protocols, eight saliva samples were obtained during the course of the experiment (Salivette® Cortisol - Sarstedt, Nümbrecht, Germany): at median times of −60 (arrival), −2, 5, 12, 21, 28, 33 and 38 minutes relative to the onset of the first stress period. Concentration of salivary free cortisol was measured using a commercially available chemiluminescence-immuno-assay (IBL, Hamburg, Germany).

### Cognitive Performance

We used the number of questions attempted, the number answered correctly and the mean time taken to answer questions to assess cognitive performance during the stress task.

### Statistical Analysis

Analyses were carried out using Stata 11 (StataCorp, College Station, Texas, USA). If necessary, variables were log transformed prior to parametric testing. Associations of overweight/obesity (BMI≥25) with potential confounders were assessed with logistic regression. Cardiovascular responses to stress were calculated by subtracting initial resting values from mean values during stress. Comparisons of values at rest with those during stress and recovery and comparisons between the two stress periods were performed using random-effects generalised least squares modelling. Cortisol responses were calculated by subtracting the resting level just prior to stress onset from the peak value (28 minutes after stress began). Relationships between BMI and stress-response variables were assessed using multiple linear regression. To illustrate these associations, comparisons of stress responses were made between overweight/obese and normal weight participants, using random-effects generalised least squares modelling. Partial correlation was used to compare the relative strength of associations between BMI and different types of adiposity and to assess the relative contribution of visceral and subcutaneous adiposity to stress responsiveness. All of these models were adjusted routinely for the age and sex of participants. These models were all repeated with social class, ethnicity, education, physical activity, tobacco and alcohol consumption, and chronic stress exposure as additional covariates to test for possible confounding effects.

## Results

### Participant Characteristics

Participants were predominantly white European, well-educated non-smokers (Table 1). Their BMI ranged from 19.1 to 42.2 and 49 participants (56%) were overweight/obese. Obesity was associated with being female, older, white European, less well educated, less physically active and drinking alcohol. Smoking, stress exposure in the preceding month and social class were not associated with overweight/obesity.

**Table 1:** Participant characteristics.

	N (%) or median (range)	Odds Ratio of Overweight/Obesity	*P*-value
Male Sex	43 (49%)	0.95	0.03
Age (Years)	38.3 (18.5–45.7)	1.07	0.03
Smoker	9 (10%)	0.99	0.99
Alcohol (U.wk^−1^)	10 (0–90)	1.04	0.01
NSSEC Class 1–3	78 (92%)	1.02	0.98
White European	74 (84%)	3.88	0.04
>A-level Educated	60 (68%)	0.37	0.04
Physical Activity Index	2.8 (1.5–3.6)	0.30	0.03
Perceived Stress	16 (2–30)	0.98	0.60

### Responses to the MIST

During the MIST, participants achieved a median of 48% (IQR 45–49%) correct answers, but the number of questions answered (21–70) and the number answered correctly (3–35) varied widely, showing that the MIST successfully calibrated apparent performance for a wide variation in ability.

**Figure 2:**
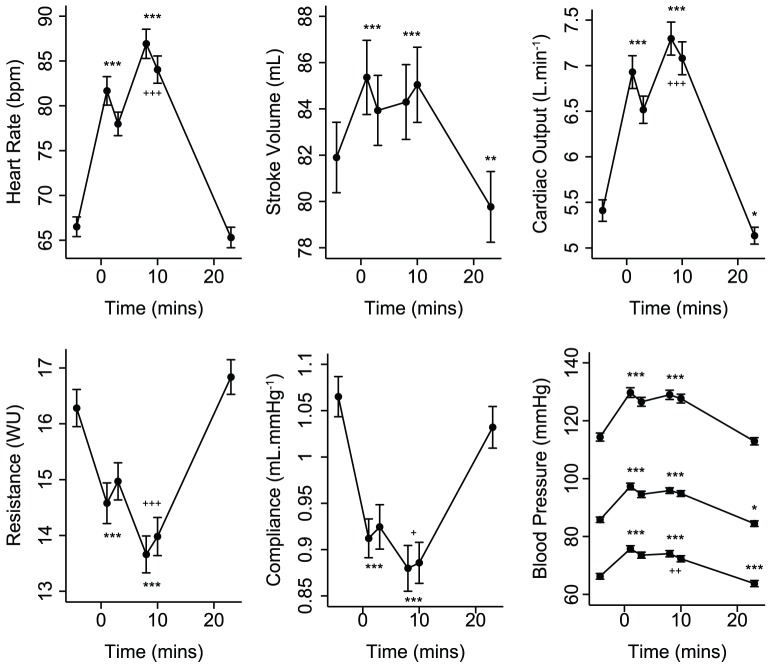
Mean (±SE) cardiovascular response to stress. ^*^ *P*<0.05, ^**^ *P*<0.01, ^***^ *P*<0.001 for random-effects generalised least squares comparisons of mean values during stress and recovery with initial resting values. ‘+’ symbols refer to similar comparisons between the two stress periods.

**Figure 3:**
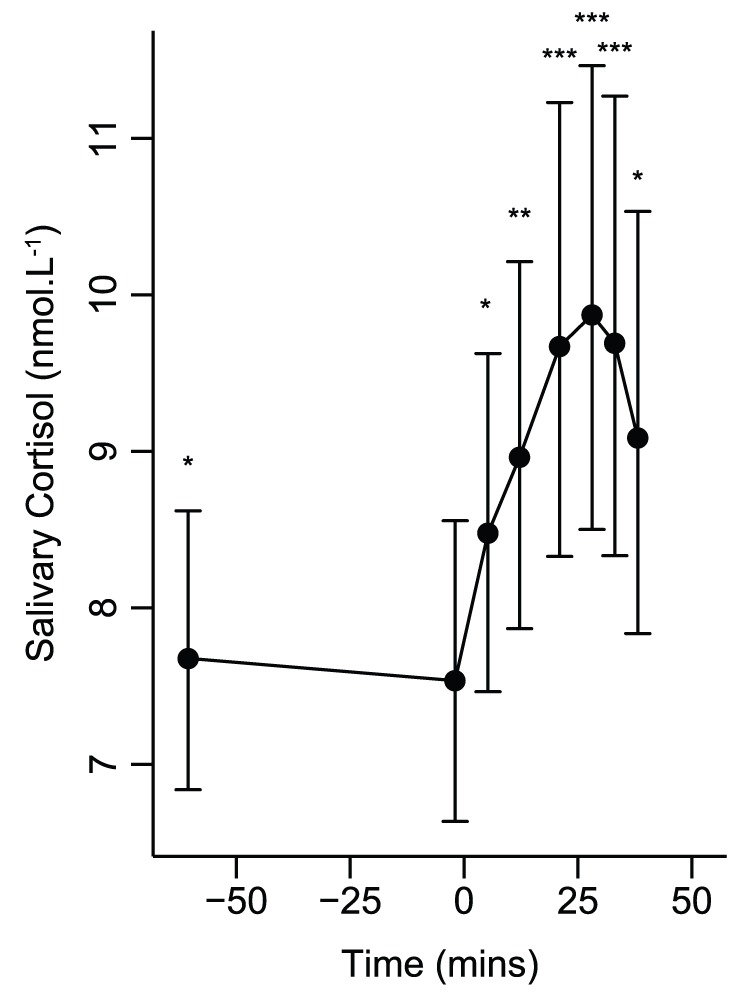
Geometric mean (±geometric 95% CI) salivary cortisol response to stress. ^*^ *P*<0.05, ^**^ *P*<0.01, ^***^ *P*<0.001 for random-effects generalised least squares comparisons of values with those at rest.

The MIST provoked a strong cardiovascular response (Figure 2) characterised by increased HR, BP, SV, and CO, and decreased TPR and arterial compliance. The second stress period stimulated significant further HR, CO, TPR and compliance responses. During recovery, HR, TPR, compliance and SBP did not differ from resting levels but SV, CO, MBP and DBP were significantly lower. The MIST also caused a significant salivary cortisol increase; peaking 28 minutes after stress began (Figure 3).

### Associations of Mental Stress Responses with BMI

**Figure 4:**
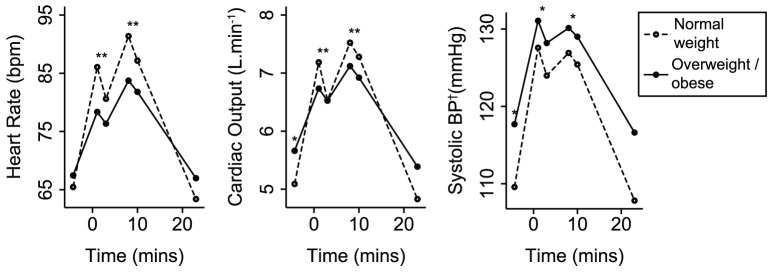
Mean cardiovascular response to stress, comparing overweight/obese and normal weight participants. Three key response variables are shown. Random-effects generalised least squares regression was used to assess differences. ^*^ *P*<0.01, ^**^ *P*<0.0001. At rest, *P*-values show the significance of the difference between groups. During stress, *P*-values refer to comparisons of the response to stress with respect to rest. Values during recovery did not differ significantly from resting values. ^†^Blood pressure.

**Figure 5:**
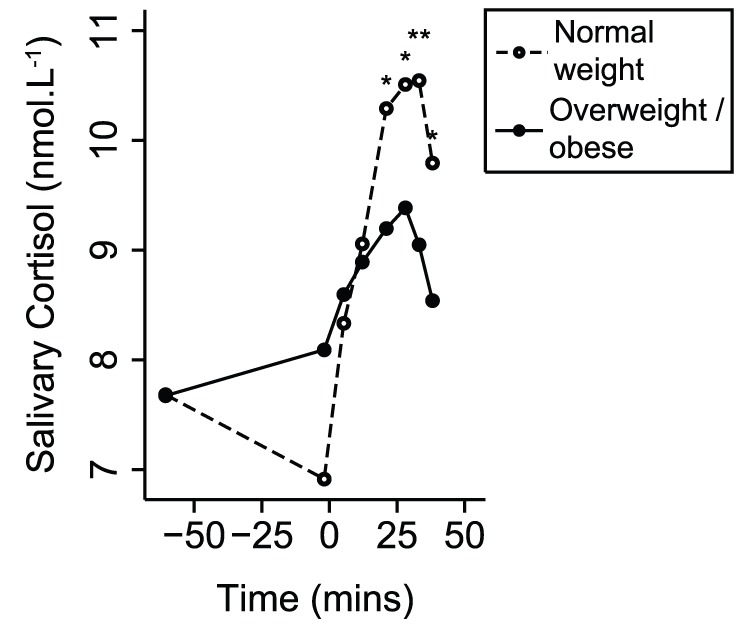
Geometric mean salivary cortisol response to stress, comparing overweight/obese and normal weight participants. Random-effects generalised least squares regression was used to assess differences. ^*^ *P*<0.01, ^**^ *P*<0.001. *P*-values refer to comparisons of the response to stress with respect to rest.

At rest, greater BMI was only associated with increased BP. However, during the MIST, greater BMI was associated with smaller increases of HR, SV, CO and BP and a smaller decrease of TPR (Table 2). There was no significant association of BMI with arterial compliance response. The different responses of HR, CO and systolic BP for overweight/obese and normal weight participants are illustrated in Figure 4. The differences of HR depended largely on their lower peak values. However, the differences of CO and SBP depended partly on higher resting values. Nevertheless, associations of BMI with HR, CO and SBP responses persisted after adjustment for resting levels. Greater BMI was associated with lower cortisol stress response (r = −0.25, *P* = 0.04), but not with resting cortisol (r = 0.07, *P* = 0.55). On average, overweight/obese participants demonstrated a significantly lower cortisol stress response than normal weight participants (Figure 5). Greater BMI was also associated with fewer attempted questions (r = −0.24, *P* = 0.03), fewer answered correctly (r = −0.28, *P* = 0.01) and greater time taken to answer questions (r = 0.26, *P* = 0.02). On average, participants who were overweight/obese attempted 5.3 fewer questions (*P* = 0.01), got 3.3 fewer questions right (*P* = 0.004) and took 0.7 seconds longer to answer them (*P* = 0.04), compared to normal weight participants.

**Table 2:** Linear regressions (ß) of BMI on cardiovascular parameters at rest and their changes in response to stress.

	Rest	Δ Stress 1	Δ Stress 2	Δ Recovery
HR (bpm.unit^−1^)	0.17	−0.50^**^	−0.51^*^	0.08
SV (mL.unit^−1^)	0.32	−0.33^*^	−0.42^**^	−0.15
CO (mL.min^−1^.unit^−1^)	39	−61^**^	−74^**^	−2
TPR (WU.unit^−1^)	−0.054	0.108^*^	0.132^*^	0.075
Compliance (mL.mmHg^−1^.unit^−1^)	−0.0038	0.0034	0.0028	0.0051
SBP (mmHg.unit^−1^)	0.81^**^	−0.41^*^	−0.32^*^	−0.14
MBP (mmHg.unit^−1^)	0.43^*^	−0.31^**^	−0.29^**^	0.06
DBP (mmHg.unit^−1^)	0.28	−0.19	−0.14	0.09

Changes for the stress and recovery periods were calculated with respect to rest. Associations were adjusted for age and sex. Note that mean TPR and compliance fell in response to stress. Therefore, positive associations of these two measures with BMI indicate smaller responses. ^*^ *P*<0.05, ^**^ *P*<0.01.

### Associations of Mental Stress Responses with Fat Distribution

We found that visceral adiposity was more strongly correlated with BMI (r = 0.63, *P*<0.001) than subcutaneous adiposity was (r = 0.47, *P*<0.001) and that visceral adiposity did not correlate with subcutaneous adiposity (r = −0.05, *P* = 0.64). The relative importance of subcutaneous and visceral adiposity was examined using these measures together as explanatory variables in multiple linear regression models (Table 3). At rest, visceral adiposity was the stronger determinant of greater resting HR and BP. More importantly, these models also showed that visceral adiposity was the principal determinant of reduced HR, CO and BP stress responses and subcutaneous adiposity was the dominant factor in reduced TPR response. Neither fat compartment was a stronger determinant of SV responses, suggesting similar contributions to the association of BMI with reduced SV response. In further multiple regression models, only visceral adiposity was associated with reduced cortisol response (r = −0.29, *P* = 0.02). Neither fat compartment was more strongly associated with cognitive performance.

**Table 3:** Partial correlations (r) of adiposity measures with cardiovascular parameters at rest and their changes in response to stress.

	Adipose Tissue	Rest	Δ Stress 1	Δ Stress 2	Δ Recovery
HR	Subcutaneous	−0.00	−0.06	−0.07	−0.10
	Visceral	0.22*	−0.24*	−0.19	0.10
SV	Subcutaneous	0.10	−0.08	−0.17	−0.07
	Visceral	−0.16	−0.17	−0.04	−0.18
CO	Subcutaneous	0.04	−0.10	−0.07	−0.12
	Visceral	0.19	−0.28*	−0.24*	0.01
TPR	Subcutaneous	−0.15	0.26*	0.28*	0.17
	Visceral	0.06	0.07	0.10	−0.09
Compliance	Subcutaneous	−0.07	−0.01	0.19	0.03
	Visceral	−0.18	0.11	−0.06	0.08
SBP	Subcutaneous	0.01	−0.02	−0.16	−0.02
	Visceral	0.27*	−0.21	−0.07	−0.19
MBP	Subcutaneous	−0.12	0.03	0.01	−0.02
	Visceral	0.33**	−0.27*	−0.28*	−0.06
DBP	Subcutaneous	−0.21	−0.09	0.01	−0.08
	Visceral	0.35*	−0.11	−0.25*	0.09

* *P*<0.05,

** *P*<0.01. Associations were adjusted for age and sex.

These findings were independent of potential confounders such as ethnicity, social class, education, chronic stress exposure, physical activity, and alcohol and tobacco consumption. Further adjustment for cortisol responses showed that associations of adiposity with cardiovascular stress responsiveness were also independent of neuroendocrine responsiveness.

## Discussion

We found that greater BMI was associated with generalised blunting of cardiovascular, cognitive and endocrine responses to AMS. Visceral adiposity was the dominant factor associated with the reduced neuroendocrine and cardiovascular responses to AMS. However, both visceral and subcutaneous adiposity were associated with reduced cognitive performance.

In contrast to previous studies, we used novel MR techniques to characterise fat distribution and cardiovascular responses to AMS. Cardiovascular MR (CMR) is the reference standard method for measuring CO, with significant advantages over other techniques such as impedance cardiography and Doppler ultrasound. However, traditional CMR is unsuitable for mental stress studies because data acquisition is too slow to characterize cardiovascular responses to AMS. We used accelerated CMR, which is sufficiently fast for stress studies and is still accurate and reliable [9]. With fat-water separation methods like T_2_-IDEAL, MR also offers a more accurate assessment of body fat distribution. This is because these techniques measure the proportion of fat in each spatially localized voxel [19], which can be summed to directly assess visceral and subcutaneous adiposity. BMI, waist-hip ratio and other surrogate measures of adiposity assess fat distribution less accurately.

These advanced methods were needed to address the controversy over the association between obesity and stress responsiveness. Several studies have shown positive associations between obesity and cardiovascular stress responses [5], [6], [7]. This led to the suggestion that obesity and AMS may be steps in a sequence linking adverse environments with cardiovascular morbidity. However, other studies have found the opposite or no association [20], [21], [22]. In fact, the largest study to date found that obesity was associated with reduced HR responses to AMS, although associations with BP were less clear [4].

We have also shown that obesity is associated with a reduced HR response, as well as reduced SV, CO, TPR and BP responses. Furthermore, we found that adiposity was linked to worse cognitive performance during stress and to lower cortisol responses. Together, these findings point to a more central deficit in the stress response than has been suggested by previous studies. One possible explanation for these findings is that reduced stress responsiveness is a feature of a condition that predisposes to obesity. For example, a passive coping style in rodents under stress is not only associated with reduced physiological stress responses, but also with increased insulin resistance and visceral obesity [23]. This is supported further by studies showing that reduced HR and adrenaline responses to AMS in humans predict the development of obesity up to 18 years later [8], [22]. Another possibility is that these findings are related to long-term overexposure to stressful stimuli. This can lead to under-responsiveness of stress systems [24] as well as increased appetite [25], reduced propensity to exercise [26], and obesity [2], [27]. In this study, adjustment for stress exposure in the month preceding the test did not substantially alter our results. However, our measure of chronic stress may not sufficiently reflect longer-term stress exposure and cannot, therefore, exclude stress exposure as an explanation for our results. The final possibility is that visceral fat is associated with resilience to stress, possibly mediated by secretion of chemical messengers. For example, leptin circulates in proportion to body fat and has been shown to inhibit responses to AMS [28]. These possibilities could be tested in future longitudinal studies using our methods, where the relative timing of stress exposure and changes in adiposity can be assessed.

The generalized stress response exists for a reason, probably conferring survival advantages from an evolutionary point of view [29], [30]. In modern humans this translates into a greater ability to deal mentally and physically with stressful situations. Thus, it is possible that adiposity, particularly of the visceral compartment, may be associated with a reduced ability to cope with stressful situations. However, greater cardiovascular responses to AMS (particularly of BP) are associated with cardiovascular morbidity [4]. Thus, our results might imply that adiposity could protect against negative cardiovascular effects of stress. In our study, reduced stress responses were predominantly related to visceral fat, which is strongly associated with known risk factors for cardiovascular disease such as impaired glucose tolerance, insulin resistance, dyslipidaemia [31], [32], and atherosclerosis [33], [34]. Therefore, any benefit conferred is likely to be overwhelmed by detrimental effects of visceral adiposity.

Interestingly, visceral adiposity was not the dominant factor in all of our findings. We found that subcutaneous adiposity was more strongly associated with blunted reductions of TPR than visceral adiposity was. The reasons for this disparity are not clear but suggest the possibility of different mechanisms for the effects of the two adipose tissue types. In normal weight individuals, resistance arterioles in subcutaneous fat dilate substantially in response to acetylcholine. There is evidence that this mechanism is impaired in the obese [35]. Thus, our finding may point to a failure of vasodilatation in subcutaneous adipose tissue.

### Limitations

A modest population size might be considered the main limitation of our study. Although many of our findings were strongly statistically significant, larger studies using our methods should be considered in future to allow for detailed sub-group analyses by, for example, sex or age groups. Larger studies have previously been needed to show associations between surrogate measures of adiposity and less accurate measures of cardiovascular function. However, we believe our study demonstrates that fewer participants are needed when highly accurate measures of cardiovascular function and adiposity are used. Furthermore, our population was carefully selected to exclude disease and there was significant variation in adiposity. We also characterised and controlled for a broad range of potential confounders, including activity levels and recent stress exposure, although we could not assess long-term stress exposure. Variation of social class, education and ethnicity in our sample was restricted, which may limit generalizability of our results, but should also minimise the potential confounding effects of these factors. We did not measure metabolic parameters in our participants although none of them were diabetic, hypertensive or had diagnosed metabolic disorders. However, we cannot exclude the possibility that variations of metabolic function might explain associations between obesity and stress responsiveness. Future studies might consider blood sampling to assess metabolic function. This should be done on a separate visit because blood sampling affects AMS and AMS affects metabolic function. Our study describes cross-sectional associations and cannot, therefore, determine causal pathways. We have proposed several possibilities but further studies are required to test them.

### Conclusion

We found that adiposity was associated with a generalised blunting of cardiovascular, neuroendocrine and cognitive responses to AMS. We believe this is evidence of centrally reduced stress responsiveness, but we can only speculate about possible explanations. Cardiovascular and neuroendocrine under-responsiveness were predominantly related to increased visceral adiposity. The consequence of reduced responsiveness may be a worse ability to cope with challenging situations. This possibility requires further study, but it suggests that maintaining normal weight may have performance-related benefits in addition to known benefits for cardiovascular health.

## References

[1] Calle EE, Thun MJ, Petrelli JM, Rodriguez C, Heath CW (1999). Body-mass index and mortality in a prospective cohort of U.S. adults.. N Engl J Med.

[2] Wardle J, Chida Y, Gibson EL, Whitaker KL, Steptoe A (2011). Stress and adiposity: a meta-analysis of longitudinal studies.. Obesity.

[3] Yusuf S, Hawken S, Ounpuu S, Dans T, Avezum A (2004). Effect of potentially modifiable risk factors associated with myocardial infarction in 52 countries (the INTERHEART study): case-control study.. Lancet.

[4] Chida Y, Steptoe A (2010). Greater cardiovascular responses to laboratory mental stress are associated with poor subsequent cardiovascular risk status: a meta-analysis of prospective evidence.. Hypertension.

[5] Pasquali R, Anconetani B, Chattat R, Biscotti M, Spinucci G (1996). Hypothalamic-pituitary-adrenal axis activity and its relationship to the autonomic nervous system in women with visceral and subcutaneous obesity: effects of the corticotropin-releasing factor/arginine-vasopressin test and of stress.. Metabolism.

[6] Tabara Y, Kohara K, Nakagawa S, Handa J, Hayashi M (2008). Effects of obesity and smoking on mental stress-induced blood pressure and augmentation index responses in normotensive young males: the J-SHIPP study.. Hypertens Res.

[7] Waldstein SR, Burns HO, Toth MJ, Poehlman ET (1999). Cardiovascular reactivity and central adiposity in older African Americans.. Health Psychol.

[8] Carroll D, Phillips AC, Der G (2008). Body mass index, abdominal adiposity, obesity, and cardiovascular reactions to psychological stress in a large community sample.. Psychosom Med.

[9] Jones A, Steeden JA, Pruessner JC, Deanfield JE, Taylor AM (2011). Detailed assessment of the hemodynamic response to psychosocial stress using real-time MRI.. J Magn Reson Imaging.

[10] Kowalik G, Steeden JA, Atkinson D, Muthurangu V (2011). A networked GPU reconstructor within the clinical workflow for rapid fat quantification.. Proceedings of the 19th Annual Meeting of ISMRM.

[11] (2012). Office for National Statistics. The National Statistics Socio-economic Classification.. Accessed 3 Jan.

[12] Baecke JA, Burema J, Frijters JE (1982). A short questionnaire for the measurement of habitual physical activity in epidemiological studies.. Am J Clin Nutr.

[13] Jacobs DR, Ainsworth BE, Hartman TJ, Leon AS (1993). A simultaneous evaluation of 10 commonly used physical activity questionnaires.. Med Sci Sports Exerc.

[14] Cohen S, Kamarck T, Mermelstein R (1983). A global measure of perceived stress.. J Health Soc Behav.

[15] Dickerson SS, Kemeny ME (2004). Acute stressors and cortisol responses: a theoretical integration and synthesis of laboratory research.. Psychol Bull.

[16] Steeden JA, Atkinson D, Hansen MS, Taylor AM, Muthurangu V (2011). Rapid flow assessment of congenital heart disease with high-spatiotemporal-resolution gated spiral phase-contrast MR imaging.. Radiology.

[17] Stergiopulos N, Meister JJ, Westerhof N (1994). Simple and accurate way for estimating total and segmental arterial compliance: the pulse pressure method.. Ann Biomed Eng.

[18] Yu H, McKenzie CA, Shimakawa A, Vu AT, Brau AC (2007). Multiecho reconstruction for simultaneous water-fat decomposition and T2* estimation.. J Magn Reson Imaging.

[19] Molarius A, Seidell JC (1998). Selection of anthropometric indicators for classification of abdominal fatness: a critical review.. Int J Obes.

[20] Hamer M, Boutcher YN, Boutcher SH (2007). Fatness is related to blunted vascular stress responsivity, independent of cardiorespiratory fitness in normal and overweight men.. Int J Psychophysiol.

[21] Steptoe A, Wardle J (2005). Cardiovascular stress responsivity, body mass and abdominal adiposity.. Int J Obes.

[22] Flaa A, Sandvik L, Kjeldsen SE, Eide IK, Rostrup M (2008). Does sympathoadrenal activity predict changes in body fat? An 18-y follow-up study.. Am J Clin Nutr.

[23] Boersma GJ, Benthem L, van Dijk G, Steimer TJ, Scheurink AJ (2010). Coping style predicts the (in)sensitivity for developing hyperinsulinemia on a high fat diet in rats.. Physiol Behav.

[24] Juster RP, Sindi S, Marin MF, Perna A, Hashemi A (2011). A clinical allostatic load index is associated with burnout symptoms and hypocortisolemic profiles in healthy workers.. Psychoneuroendocrinology.

[25] Groesz LM, McCoy S, Carl J, Saslow L, Stewart J (2011). What is eating you? Stress and the drive to eat.. Appetite.

[26] Kouvonen A, Kivimaki M, Cox SJ, Cox T, Vahtera J (2005). Relationship between work stress and body mass index among 45,810 female and male employees.. Psychosom Med.

[27] Dallman MF (2010). Stress-induced obesity and the emotional nervous system.. Trends Endocrinol Metab.

[28] Heiman ML, Ahima RS, Craft LS, Schoner B, Stephens TW (1997). Leptin inhibition of the hypothalamic-pituitary-adrenal axis in response to stress.. Endocrinology.

[29] Joels M, Pu Z, Wiegert O, Oitzl MS, Krugers HJ (2006). Learning under stress: how does it work?. Trends Cogn Sci.

[30] Lupien SJ, Wilkinson CW, Briere S, Menard C, Ng Ying Kin NM (2002). The modulatory effects of corticosteroids on cognition: studies in young human populations.. Psychoneuroendocrinology.

[31] Pouliot MC, Despres JP, Nadeau A, Moorjani S, Prud'Homme D (1992). Visceral obesity in men. Associations with glucose tolerance, plasma insulin, and lipoprotein levels.. Diabetes.

[32] Despres JP (1998). The insulin resistance-dyslipidemic syndrome of visceral obesity: effect on patients' risk.. Obes Res.

[33] Nakamura T, Kobayashi H, Yanagi K, Nakagawa T, Nishida M (1997). Importance of intra-abdominal visceral fat accumulation to coronary atherosclerosis in heterozygous familial hypercholesterolaemia.. Int J Obes.

[34] Lakka TA, Lakka HM, Salonen R, Kaplan GA, Salonen JT (2001). Abdominal obesity is associated with accelerated progression of carotid atherosclerosis in men.. Atherosclerosis.

[35] Georgescu A, Popov D, Constantin A, Nemecz M, Alexandru N (2011). Dysfunction of human subcutaneous fat arterioles in obesity alone or obesity associated with Type 2 diabetes.. Clin Sci (Lond).

